# Sustainability Views and Intentions to Reduce Beef Consumption: An International Web-Based Survey

**DOI:** 10.3390/foods14152620

**Published:** 2025-07-26

**Authors:** Maria A. Ruani, David L. Katz, Michelle A. de la Vega, Matthew H. Goldberg

**Affiliations:** 1 Curriculum, Pedagogy and Assessment, Institute of Education, Faculty of Education and Society, University College London, London WC1H 0AL, UK; 2 The Health Sciences Academy, London SW6 5UA, UK; 3 True Health Initiative, The Health Sciences Academy, London SW6 5UA, UK; 4 Morse College, Yale University, New Haven, CT 06511, USA; 5 Yale Program on Climate Change Communication, Yale University, New Haven, CT 06511, USA

**Keywords:** food choice, sustainable food systems, consumer behavior, beef overconsumption, beef reduction, food perception, planetary health, environmental health literacy, climate action, dietary change

## Abstract

The environmental detriments of the growing global production and overconsumption of beef, including greenhouse gas emissions, deforestation, and biodiversity loss, are well-documented. However, public awareness of how dietary choices affect the environment remains limited. This study examines sustainability views on beef consumption and the potential for behavioral change as a step toward more sustainable intake levels. An observational web-based survey was conducted (*n* = 1367) to assess respondents’ current beef intake frequency, views on beef consumption related to planetary health, tropical deforestation, greenhouse gas emissions, and climate change, and willingness to modify beef consumption behavior. Chi-square tests were used for group comparisons, and weighted average scores were applied to rank levels of resistance to reducing beef intake. Environmental concern related to beef consumption was associated with greater beef cutback intentions and lower long-term intake reduction resistance amongst beef eaters. Beef eaters who strongly agreed that global beef consumption negatively impacts the environment were considerably more likely to express intentions to reduce their long-term beef intake compared to those who strongly disagreed (94.4% vs. 19.6%). Overall, 76.6% of beef eaters indicated wanting to eat less beef or phase it out entirely (30.7% reduce, 29.4% minimize, 16.6% stop), with only 23.4% of them intending to keep their consumption unchanged. Compelling messages that help translate awareness into action, such as the #NoBeefWeek concept explored in this study, may support individuals in adopting more sustainable food choices. These cross-national findings provide evidence for a ‘knowledge–intent’ gap in sustainable diet research, with relevance for health communicators and policymakers. Future research could examine the factors and motivations influencing decisions to modify beef consumption, including the barriers to achieving sustainable consumption levels and the role of suitable alternatives in facilitating this transition.

## 1. Introduction

The increasing production and consumption of beef are recognized as major contributors to Earth-wide environmental degradation and climate change through greenhouse gas emissions, deforestation, and biodiversity loss [1,2,3]. The livestock sector, particularly beef, is a primary driver of these issues, making dietary changes a critical area of focus for mitigating global warming and promoting sustainability. Studies have shown that beef production requires more land and water, and generates higher greenhouse gas emissions, compared to other animal protein sources [4,5]. Globally, livestock production is linked to 14.5% to 18% of all human-induced greenhouse gas emissions [2,6], with beef cattle being the largest single source, accounting for 41% of the sector’s emissions [7,8].

Supporting these sustainability concerns, a comprehensive meta-analysis involving 570 studies from 119 countries found that beef consumption has the highest environmental impact among 40 food items that together provide 90% of global protein and calories [9]. This also reflects health risks: according to the Global Burden of Disease (GBD) reports, diets high in red meat were associated with approximately 900,000 deaths worldwide in 2019 [10,11,12]. Furthermore, in 2015, the WHO’s International Agency for Research on Cancer (IARC) categorized red meat as a Group 2A carcinogen, indicating that it is probably carcinogenic to humans [13]. Highlighting the need for change, the 2019 EAT-Lancet Commission identified reducing global red meat consumption, including beef, by more than 50% as a critical strategy benefiting both humans and the planet [14].

The adverse effects of beef production practices on critical ecosystems, such as the Amazon rainforest, have also received considerable attention by major media outlets (e.g., [15,16,17,18,19,20,21,22,23]). Despite this extensive coverage and overwhelming scientific evidence connecting global meat supply to environmental damage, people continue to adhere to meat-rich diets, in part due to a lack of understanding of the repercussions [24,25,26,27,28], suggesting that the public may not fully recognize their own dietary choices (and associated demand) as contributing to the problem.

Reducing beef consumption to sustainable levels presents significant challenges. In China, East Asia, and other middle-income regions, beef consumption continues to be on the rise [26,29,30,31]. According to the OECD-FAO Agricultural Outlook 2025–2034 report [32], global beef demand is projected to increase by 13% by 2034, driven largely by middle- and high-income countries. Meanwhile, lower-income regions pose environmental efficiency concerns, with Sub-Saharan Africa’s beef cattle herds being three times larger than those in North America yet yielding only one-tenth the output per animal [32]. Although some reductions are occurring in the UK and US, the current rate of change seems insufficient [33,34], and the US remains one of the largest per capita red meat consumers in the world [35].

Following the UK Climate Change Committee’s call for a 20% reduction in beef, lamb, and dairy intake per person by 2030 to support the country’s net zero emissions targets for 2050 [36], a survey of 2215 UK adults revealed that stronger beliefs in the health benefits of eating less meat and the importance of protecting the environment were associated with higher acceptability of meat reduction policies [34]. Still, over 40% of adults are thought to exceed the UK recommended intake limit of no more than 70 g of red or processed meat a day [34,37].

Cultural norms and resistance to change have perpetuated beef as a staple in diets, even in contexts where environmental awareness is heightened (e.g., beef served at the United Nations’ climate change COP meetings) [38,39,40]. Additionally, disproportionate beef consumption seems prevalent among certain demographics, such as males and older adults in the US [33,41], denoting pronounced social differences in dietary habits and norms. Barriers such as taste and texture, perceived nutritional value, associations with strength and affluence, food environment, meat industry influence and advertising, and persistent pro-meat misinformation may also hinder people’s willingness to reduce their beef consumption [27,28,42,43,44,45,46,47,48,49,50,51,52].

While the environmental impacts of global beef production are widely acknowledged, there could be a disconnect between general awareness of these issues and the recognition that individual consumption may contribute to them. Many people might understand the cumulative environmental damage caused by beef production practices but may not fully connect their personal dietary choices with ecological effects. This potential rift between awareness and individual responsibility could represent a significant barrier to encouraging meaningful reductions in beef demand and, consequently, achieving a more sustainable supply [53].

Understanding the public’s views on environmental issues and long-term intentions regarding beef consumption can be informative when developing communication strategies and policies aimed at supporting the adoption of more sustainable diets. This context forms the basis for our exploratory analysis of public perceptions of beef consumption and self-reported beef reduction intentions.

An observational web-based survey was conducted to assess respondents’ current beef intake frequency, views on beef consumption in relation to planetary health, tropical deforestation, greenhouse gas emissions, climate change, and human health, willingness to modify beef consumption behavior for just a week under a hypothetical #NoBeefWeek concept, and long-term change intentions regarding beef intake. Based on self-reported data, the current study aims to explore:

1. The respondents’ views about the impact of beef consumption on human health and the environment, and the connection between the ecological perspectives of beef eaters and their long-term change intentions in relation to beef intake. *(How do respondents perceive the impact of beef consumption on health and the environment, and how do these views relate to their long-term intake intentions?)*

2. The respondents’ rates of acceptance or hesitancy towards the postulated #NoBeefWeek concept, their potential perceived difficulty in temporarily avoiding preferred beef products, and their likelihood of encouraging others to stop purchasing or eating beef products for a week. *(How willing are respondents to avoid beef products for one week, and how likely are they to face challenges or encourage others to do the same?)*

3. The long-term change intentions (if any) of beef eaters regarding their consumption of beef products, and the relationship between self-reported beef intake frequencies and the levels of willingness or resistance to limit or phase out beef from the diet over the long haul. *(What are the long-term intentions of beef eaters regarding maintaining, reducing, or stopping beef consumption, and how is current intake frequency associated with these intentions?)*

This work begins by describing the survey methodology and participant demographics, followed by an analysis of themes related to beef consumption and environmental concern. It concludes with a discussion of the implications for sustainable diet communication and potential avenues for future research.

## 2. Materials and Methods

### 2.1. Study Design and Population

An anonymous online survey questionnaire was developed to assess respondents’ environmental views on beef consumption, their reactions to the hypothetical #NoBeefWeek proposal, and the long-term consumption change intentions among those who eat beef. A non-probabilistic, opportunity sampling method was applied, consistent with the study’s exploratory nature and facilitated by the distribution of the survey questionnaire amongst over 100,000 email subscribers of The Health Sciences Academy [54], who represent a demographic of higher-than-average education and keenness for nutritional matters (primarily from the United Kingdom, the United States, and Australia, among circa 170 other countries), and approximately 11,000 email subscribers (mainly from the United States) of its social responsibility program, True Health Initiative [55]. This was combined with some social media shares and webpages to encourage survey participation, with responses collected between 9 December 2021 and 11 April 2022, during the mid-pandemic period, marked by widespread societal and dietary adjustment [56].

A total of 1394 respondents participated in the survey. Following this, 10 minors (respondents aged 17 or under) were removed from the analysis. Out of the 1384 participating adults, 1367 provided informed consent for their anonymized responses to be included in the analysis. Consequently, the final sample for this study comprised 1367 respondents.

### 2.2. Ethical Considerations

Ethical approval was obtained under the Ethics Review Procedures at the Institute of Education, University College London, with data registration number Z6364106/2018/06/67. The survey design, distribution, and data processing were in accordance with the approval guidelines.

Survey participation was voluntary, with no financial compensation and no known risks for participating or not participating. Informed consent was obtained electronically via the initial page of the online survey, and participants were free to skip questions or exit the questionnaire at any time without consequences. Responses were captured online through a web-based form created on the SurveyMonkey platform and analyzed anonymously. Survey configuration settings were adjusted upfront to restrict participants from taking the survey more than once and to collect responses without having to formally submit the form. As participation was entirely voluntary and uncompensated, no attention check items were included. The survey was accessible through a web link and could be filled out on any Internet-connected computer or device and browser.

### 2.3. Instrument Measures and Outcomes

The survey instrument developed for this research study was a split-logic questionnaire in English, translated to 8 languages (Spanish, French, Portuguese, Italian, Russian, Arabic, Chinese, and Hindi), consisting of a voluntary consent question on the front page, followed by 12 main questions (including 14 sub-questions) in 4 main parts, as described below.

#### 2.3.1. General Demographics and Characteristics

This first part collected participant data on country of residence, age, gender, current diet, beef intake frequency, interest in beef consumption and planetary health from having completed the survey, and nutrition or health profession involvement or lack thereof.

Supplementary Table S1 describes the socio-demographic characteristics of respondents, with a predominance of women (73.1%), which is likely a reflection of the demographics of The Health Sciences Academy and True Health Initiative and the general predisposition for females to be considerably more likely than males to participate in fully voluntary online surveys, including those related to diet and health [57,58]. A total of 84 countries from five continents were represented, with 27% of respondents being from the United Kingdom, 27% from the United States, 9.1% from India, 5.3% from Canada, 3% from Australia, 2.9% from France, 1.9% from Spain, and 23.8% from another 77 countries. The study had diverse age representation, as follows: 25.3% aged 18–35, 23.6% aged 36–45, 34.6% aged 46–60, and 16.0% aged 61 and above.

When asked about their current diet, the majority stated that they eat animal foods (81%) in different proportions, with about 7 in 10 of them (71.4%) also consuming beef products at varying frequencies, most commonly from three times a week to twice a month (87% of beef eaters). Respondents were also asked whether their interest in the planetary health effects of beef consumption had increased from completing the survey, and nearly 7 in 10 (67.9%) said it had. Lastly, although 40.8% had no involvement in the nutrition or health profession, 43.1% affirmed being a nutrition or health professional, and 16.2% said that they were studying for a career in these fields. Additional participant characteristics are detailed in Supplementary Table S1.

#### 2.3.2. Acceptance and Feasibility of the #NoBeefWeek Concept

The second part of the survey gauged the participants’ willingness to modify their beef consumption behavior for one week as part of the proposed #NoBeefWeek concept and their likelihood of encouraging others to temporarily stop purchasing or eating beef products during that week. Self-stated levels of potential difficulty in avoiding different beef products for at least a week were also assessed, including steaks, hamburgers, meatballs, minced (ground) beef, salami, and spreads, among others.

#### 2.3.3. Views About Beef Consumption, Human Health, and the Environment

The third part of the survey consisted of five statements related to the impact of beef consumption on planetary health, deforestation, global climate change, greenhouse gas emissions, and human health, plus one other statement related to the impact of the livestock industry on global greenhouse gas emissions. These statements were informed by insights derived from a prior observational survey conducted among Sydney residents, which explored attitudes and behaviors related to meat consumption [48]. Respondents were asked to rate their agreement with each statement on a five-point Likert scale (‘strongly agree’, ‘slightly agree’, ‘neither agree nor disagree’, ‘slightly disagree’, and ‘strongly disagree’).

Searchability bias (looking up answers online prior to responding) cannot be ruled out, yet the average response time detailed above suggests that this was not likely, as it would have taken considerably longer to search for information related to the six statements and answer the rest of the survey. Further, the statements presented invited more generic views or reactions rather than completely verifiable responses.

#### 2.3.4. Long-Term Beef Consumption Change Intentions

Given that survey participation most likely encouraged contemplation of the environmental impact of consuming beef products, related dietary change intentions for the future were also measured. Thus, the fourth part of the survey was designed to evaluate the respondents’ intentions regarding long-term changes in their consumption of beef products, with the possible answer choices being to ‘stop’, ‘minimize’, ‘reduce’, or ‘keep unchanged’. The specific question asked was *“What are your long-term intentions in relation to eating beef and its derivatives?”*

### 2.4. Statistical Analysis

Statistical analyses and visualizations were performed using Microsoft^®^ Excel^®^ for Microsoft 365 MSO (Version 2503, Build 16.0.18623.20116, 64-bit), the open-access statistical program Social Science Statistics (Version 2018), and SurveyMonkey Enterprise advanced statistical analyses features, such as indicating statistical significance for cross-tabulated data. A *p*-value below 0.05 was deemed statistically significant.

Descriptive statistics were applied for the analysis of the collected data, such as numbers of observations, percentages, and means. For cross-tabulations comparing survey answer choices among different groups of respondents, weighted averages of aggregated data were calculated. To provide a ranking of the data related to beef intake frequencies, responses were weighted as follows: ‘stop my consumption’ 0, ‘minimize my consumption’ 1, ‘reduce my consumption’ 2, and ‘keep my consumption unchanged’ 3. The weighted averages were then calculated and sorted in descending order.

Descriptive statistics and chi-square comparisons were used to identify directional trends and to test for significant differences across groups. Weighted scoring and group cross-tabulations were applied to enable comparative analysis across sub-populations (e.g., intake frequency, agreement levels). This analytical approach allows for clarity in interpretation and inference.

## 3. Results

### 3.1. High Acceptance of the #NoBeefWeek Concept

Table 1 depicts the rates of acceptance and hesitancy towards the #NoBeefWeek concept, as well as respondents’ likelihood of encouraging others to temporarily stop eating or purchasing beef products if the concept were implemented. About 3 in 4 beef eaters (75.9%) said they were willing to skip beef consumption for one week upon becoming aware of the concept via the present survey. Two thirds (66.6%) of all respondents, including both beef eaters and beef avoiders, reported that they were either ‘likely’ or ‘extremely likely’ to encourage others to skip beef during #NoBeefWeek.

The vast majority of beef eaters found most of the listed beef products ‘easy’ or ‘extremely easy’ to give up for at least one week (Supplementary Table S2). Hamburgers, meatballs, and beef spreads such as pâté ranked as the easiest beef products to avoid for a week, whereas steaks, minced (ground) beef, and beef in wraps, sandwiches, sauce, stuffing, and pies ranked as the hardest. Detailed responses for each beef product can be found in Supplementary Table S2.

### 3.2. Prevalent Intentions to Reduce Beef Consumption in the Long Term

**Figure 1:**
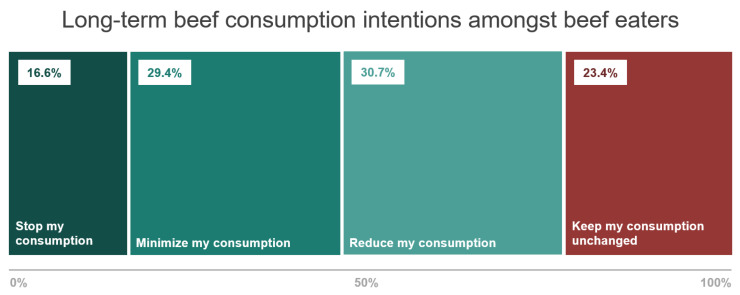
Long-term intentions to change beef consumption amongst beef eaters.

Figure 1 shows that the most prevalent long-term intention amongst beef eaters was to cut back their beef consumption. About 3 in 4 beef eaters (76.6%) indicated wanting to eat less beef or even phase it out (i.e., 30.7% reduce, 29.4% minimize, 16.6% stop), with only 23.4% of them intending to keep their consumption unchanged.

#### Greatest Resistance to Long-Term Intake Cutbacks Amongst Frequent Beef Eaters

Table 2 shows the relationship between beef intake frequencies and long-term intentions to reduce, minimize, stop, or keep beef consumption unchanged. We observed that daily beef eaters exhibited the greatest resistance to long-term beef intake cutbacks, with 67.9% of them wanting to keep their long-term consumption unchanged, and only 7.1%, 14.3%, and 10.7% intending to reduce it, minimize it, or stop it (respectively). Conversely, those who reported the lowest beef consumption frequencies were the most willing to further reduce and even phase out their intake in the long run, compared to the more regular beef eaters. For instance, 29.2% of participants consuming beef products a couple of times a month or less intended to stop their consumption altogether, 36.4% to minimize it, 20.8% to reduce it, and only 13.6% to keep it unchanged.

### 3.3. Predominance of Environmental Concern in Relation to Beef Consumption

Supplementary Table S3 details the respondents’ views about the impact of beef consumption on planetary health, deforestation, global climate change, greenhouse gas emissions, and human health as well as that of the livestock industry on global greenhouse gas emissions. Over two thirds (67.8%) of respondents agreed with all six statements presented to them in varying degrees. For example, 75.5% of respondents agreed that beef consumption negatively impacts planetary health and 69.6% that it negatively impacts human health. Other statements with a very high level of agreement include that *“Beef consumption results in more greenhouse gas emissions than plant-food consumption”* (71.5%) and “*The livestock industry is the biggest contributor of global greenhouse gas emissions from food production*” (69.8%). The statement with the lowest level of agreement was that *“Beef consumption is one of the main causes of global climate change”* (58.1%), followed by *“Beef consumption is the leading cause of deforestation in the Amazon and other tropical forests”* (62.1%).

#### Recognition of Environmental Impacts Associated with Greater Beef Cutback Intentions and Lower Change Resistance

**Figure 2:**
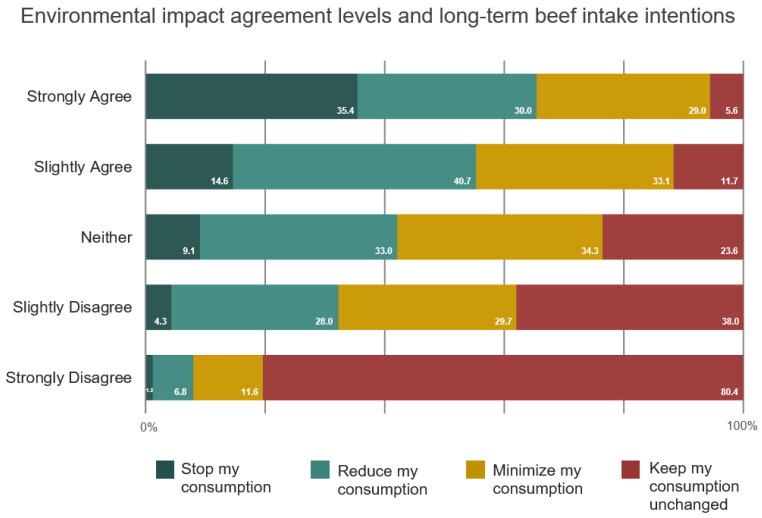
Relationship between agreement levels on the five environmental statements and long-term beef intake change intentions among beef eaters.

Although most people in the sample agreed that beef consumption and livestock production negatively impact the environment, not all beef eaters intended to cut back their own intake in the long run, with the most reluctant being those who did not agree that these foods have a detrimental effect on the environment, as shown in Figure 2. On the other hand, higher levels of agreement across all five statements that beef consumption and livestock production affect the environment were associated with reduced long-term intake change resistance amongst beef eaters.

Disagreement that beef consumption negatively impacts planetary health was associated with a lower intention to reduce one’s own intake in the long term, where 91% of strong dissenters indicated an intention to keep their beef consumption unchanged (Supplementary Table S4). A similar inclination to keep one’s own beef consumption unchanged was presented amongst strong dissenters about this being the leading cause of tropical deforestation (83.8%), one of the main causes of global climate change (70.6%), behind more greenhouse gas emissions than plant food consumption (82.4%), and the livestock industry being the biggest contributor of global greenhouse gas emissions from food production (81.9%). In contrast, intentions to limit one’s own beef intake in the long term were almost unanimous (94.4%) amongst those who strongly agreed on all five statements that beef consumption and livestock production are harmful to the environment, capturing all intake patterns from daily to occasional beef eaters. Further information about these relationships can be found in Supplementary Table S4.

## 4. Discussion

The results of our study provide valuable insights into public perceptions and intentions regarding beef consumption in relation to environmental and health concerns. The findings reveal significant acceptance of the #NoBeefWeek concept and indicate a general willingness among respondents to modify their dietary habits, particularly in the context of reducing beef consumption for environmental benefits.

### 4.1. Acceptance of the #NoBeefWeek Concept

The high acceptance rates of the #NoBeefWeek concept denote an awareness and willingness among beef consumers to potentially engage in temporary dietary changes for environmental causes. The vast majority of beef eaters expressed readiness to abstain from beef for one week, suggesting both the feasibility and potential effectiveness of targeted initiatives in mobilizing public action. This finding aligns with previous studies indicating that providing information about the environmental toll of purchasing or eating meat is associated with individuals becoming more aware and willing to reduce its consumption [25,28,59,60,61,62,63]. Saari et al. (2021) proposed that environmental concern, underpinned by knowledge and environmental risk awareness, drives behavioral intention or willingness to ‘sacrifice’ and adopt more sustainable consumption practices [64]. In addition, the high likelihood of both beef eaters and beef avoiders encouraging others to participate in the #NoBeefWeek bid reflects the potential of social influence to foster environmentally sustainable consumption.

The concept of ‘de-meatification’ and campaigns like Meatless Monday have shown potential in promoting reduced meat consumption for environmental and health benefits [65]. These initiatives encourage individuals to abstain from meat on specific days or for extended periods, aiming to lower greenhouse gas emissions and decrease the demand for resource-intensive meat products like beef. Meatless Monday, for instance, has been widely adopted in various countries and institutions, demonstrating significant potential in shifting dietary habits towards more plant-based options [66]. Such campaigns can be effective models for prospective initiatives, such as #NoBeefWeek, illustrating the potential of collective action in fostering sustainable dietary changes.

Building on this, our study flags a variation in the ease with which respondents may give up different beef products for at least a week, indicating that certain items like hamburgers, meatballs, and beef spreads may be more readily substituted or discontinued than steaks, minced (ground) beef, and beef in wraps and pies. This variation suggests that relevant campaigns should consider addressing beef products that consumers find harder to limit or eliminate, for example, by highlighting accessible alternatives for these challenging items. Participants’ higher resistance to substituting steak (compared to hamburgers) may in part reflect sensory attachment [67]. Ongoing trials focused on optimizing the sensorial attributes of restructured plant-based steaks suggest potential to overcome these barriers [68]. Additionally, identifying which beef products are easier to forgo could guide educational efforts and institutional policies (such as school or workplace menu adjustments and procurement guidelines) to facilitate smoother transitions to more sustainable dietary habits.

### 4.2. Long-Term Intentions to Reduce Beef Consumption

Our survey revealed that most beef eaters expressed intentions to eat less beef or even phase it out in the long run. This trend is promising, considering the significant environmental impacts of global beef production [9,69,70,71,72,73]. However, there remained a notable resistance among daily beef consumers, with the majority of this group intending to maintain their current consumption levels. This resistance may stem from entrenched dietary habits and sociocultural factors that prioritize beef as a staple food [28,71,74,75,76] as well as extrinsic barriers such as food environment, meat industry messaging and imaging, and widespread pro-beef misinformation [27,28,42,43,44,48,49,50,51,52].

The intricate relationship between dietary habits and environmental awareness has been the focus of several investigations. Some studies have suggested that individuals with higher environmental knowledge and environmental risk perception are more likely to adopt more sustainable dietary practices [28,45,64]. Asvatourian et al. (2018) found that health-conscious diets are associated with lower greenhouse gas emissions and more pro-environmental behaviors, though increased awareness alone did not consistently translate to dietary changes [77]. In a survey conducted among Sydney residents, 29% of respondents were aware of the detrimental effects of livestock on planetary health, yet nearly a third of these individuals continued to eat meat on a daily basis [48], emphasizing the challenge of overcoming ingrained dietary habits, even when environmental awareness is present.

Similarly, an experimental study by Wistar et al. (2022) found that regular beef eaters exhibited greater resistance to reducing their consumption when exposed to environmental messages, compared to those who ate beef once a week or less [25]. This corresponds with our findings, suggesting that a stronger attachment to beef and its habitual consumption may make these individuals less responsive to environmental appeals and more resistant to reducing their current intakes. Meat substitutes, including plant-based, microbial, and cell-cultured options, may be of particular value in meeting the fixed demands of this demographic by offering similar taste, texture, and nutritional content to beef. These alternatives could help satisfy cultural and sensory expectations while presenting more sustainable options to conventional beef products [78,79,80,81].

### 4.3. Environmental Concern and Beef Reduction Intentions

The survey results demonstrate a clear correlation between environmental concern and intentions to reduce beef consumption. Respondents who strongly agreed that beef consumption has negative environmental impacts were more likely to express intentions to cut back on beef intake. Conversely, those who disagreed with the environmental statements showed greater resistance to change. This finding supports previous research suggesting that environmental awareness or concern can influence meat reduction intentions [28,45,63,77]. However, the jump from willingness to action remains a challenge, pointing to the need for more compelling appeals [82] that not only inform but also activate sustainable behavioral changes such as reducing beef intake. Per the COM-B model’s proposition, behavioral change could be more effectively achieved when motivational drivers are supported by adequate capability and opportunity [83]. Future work could test whether providing a wider variety of plant-based options or messaging about diet-change success stories could increase the likelihood of sustained beef reduction.

Although public awareness about the importance of recycling and other sustainability behaviors is relatively high [84], many individuals remain unaware of the ecological impacts of their own dietary choices, particularly regarding the consumption of meats like beef [28,85,86,87,88,89,90,91]. Research has consistently stressed the public’s limited recognition of the environmental effects of one’s own meat consumption. For example, Truelove and Parks (2012) reported that 80% of participants mentioned driving less and 45% mentioned recycling to personally help reduce global warming, while fewer than 5% mentioned reducing meat intake [92]. More recently, a study by Leiserowitz et al. (2020) found that although more than half of a representative sample of Americans believed that the production of beef, pork, dairy, and poultry contributed at least ‘a little’ to global warming, merely 27% thought that beef production contributed ‘a lot’ [88].

This evidence, including ours, points to an urgent need for public education on these topics to clearly establish the connection between consumer demand, the practices of suppliers, and the environmental consequences that result from specific production methods, helping individuals better recognize the impact of their dietary choices in a broader context.

Beef cattle, along with other livestock, occupy over a quarter of the planet’s ice-free terrestrial area [93,94] and are implicated in land degradation, soil and water acidification and pollution, water shortages, and eutrophication [9,69,70,71,72,73]. The environmental consequences of beef production extend to deforestation in the Amazon and other tropical forests, causing irreparable biodiversity attrition and amplified carbon emissions when forests are cleared and burned. Over the past three decades, more than 780,000 square kilometers of Brazilian forest have been destroyed, leading to the loss of around 2000 species and contributing nearly half of the country’s carbon emissions [95,96]. Pasture expansion for cattle production has been the primary driver of this deforestation, accounting for 87% of the clearing [97].

Notwithstanding corporations and governments gradually committing to zero-deforestation supply chains and policies since the early 2000s, over 113,000 cattle properties in the Brazilian Amazon were involved in deforestation activities between 2010 and 2018 [95], and deforestation rates increased by 47% between 2018 and 2020 [98]. Ongoing deforestation, political loosening, and illegal practices such as deliberate forest fires and prohibited beef cattle production inside protected areas continue to pose significant challenges [98,99]. Recent investigations have suggested that global warming, in combination with deforestation and regional rainfall changes, is pushing the Amazon closer to its critical tipping point, where even minor disturbances could risk an abrupt and potentially irreversible shift to a degraded state or collapse of the ecosystem in the next two and a half decades [100].

Addressing these ongoing challenges requires not only stricter enforcement of environmental policies [101] but also increased public engagement and education to help consumers recognize how their personal choices contribute to global environmental outcomes, particularly through beef demand and subsequent supply [53].

### 4.4. Implications for Public Health and Policy

The strong intentions to reduce beef consumption among a significant portion of the sample suggest potential public health and environmental benefits if these intentions are realized. Reduced beef consumption could lead to lower greenhouse gas emissions, decreased deforestation, and improved biodiversity [9,95]. While other animal protein and fat sources have been suggested (such as poultry and fish), plant-based substitutions are likely to provide superior environmental benefits [9,102,103,104]. According to Sabaté et al. (2015), producing protein from kidney beans is much more resource-efficient than from beef, likely using 18 times less land, 10 times less water, and 9 times less fuel [91]. Importantly, public health could benefit from decreased risks associated with high red meat consumption, including beef, such as certain cancers, cardiovascular diseases, and diabetes mellitus [10,11,12,13,46,105].

Meat analogs like plant-based substitutes, cultured meat, and fermentation-derived microbial protein offer promising alternatives that could contribute to sustainability [78,79,80,81]. For example, global life cycle assessment models project that replacing a fifth of per capita beef intake with microbial protein could lower methane emissions by 11% and halve the annual deforestation rate by 2050 [106]. Given that most meat analogs are categorized as ultra-processed, controlled investigations are required to explore their impact on health markers [107], and consumer acceptance of these products needs to be better understood [108,109,110,111,112,113]. Early work in this area suggests that plant-based meat-mimicking alternatives may offer some benefits to human and planetary health alike [114,115,116,117]. Educating the public about specific plant-based sources of essential nutrients like iron, calcium, and zinc, along with fortification strategies, can help mitigate potential deficiencies that might arise from reducing beef consumption [107,118].

To capitalize on these intentions, policymakers and health organizations should develop targeted strategies (e.g., consumer education campaigns, incentive-based programs, institutional guidelines) that address barriers specific to reducing beef consumption to meet sustainability goals. Prospective initiatives such as #NoBeefWeek could serve as viable models for raising awareness and encouraging behavioral change. Further research should evaluate the short- and long-term effectiveness of such strategies and identify the most effective approaches for stimulating sustainable dietary shifts.

### 4.5. Strengths and Limitations

The present study was strengthened by the large sample size and representation of all adult age groups, although its geographical, demographic (other than age), and social diversity may not fully represent the wider population. While using an opportunity sample has its merits, such as the ease of participant recruitment, self-selection bias cannot be discarded, and the findings may not be directly generalizable. However, the large and diverse age representation provides a strong foundation for future research to explore these issues in more varied populations.

It is likely that the recruited population (namely, students and subscribers of The Health Sciences Academy and True Health Initiative) had higher levels of environmental health literacy compared to the general public. To the extent this was so, it suggests potentially greater and more important knowledge deficits in the public at large regarding links between beef demand and environmental impacts. Although this sampling strategy does not support probabilistic generalization to the global population, it allows for the analysis of a demographically diverse sub-population with a predisposition toward health and sustainability topics.

Social desirability bias was likely mitigated by the use of an anonymous web-based questionnaire in our study, which typically results in more honest responses compared to other methods such as interviews and telephone surveys [119,120,121]. This is further supported by results demonstrating the greatest disagreement on the environmental burdens of beef consumption amongst respondents who reported eating beef products more frequently, and that not all beef avoiders agreed that beef consumption or the livestock industry are ecologically harmful (Supplementary Table S5).

Despite non-response bias being a common concern in online survey studies, whereby skipped questions may affect the completeness of the data available for analysis [122], this survey had a high completion rate of 90.3%. Another recognized risk of online surveys is speeding [123]. However, the average completion time was 3 min and 13 s, indicating that fast random clicking was relatively uncommon in this sample.

As this was an observational survey, no causal relationships can be inferred between awareness or acceptance of environmental impacts and intentions to reduce beef consumption. Although change intentions regarding long-term beef consumption were measured, the reasons or motivations behind these and any resulting eating behavioral change were not. Future research could focus on exploring these factors in depth, which is essential for understanding how to generate meaningful change and promote more sustainable eating habits.

## 5. Conclusions

Political, socioeconomic, and technological efforts to mitigate climate change need to be accompanied by the engagement of the public. More awareness and acceptance are critical in motivating people to change dietary behavior for the sake of the planet [124].

The findings of this survey underline the potential feasibility for prospective initiatives like #NoBeefWeek to raise awareness and encourage short-term behavioral changes in beef consumption. The high acceptance rates observed indicate a growing public willingness to consider the environmental impacts of their personal dietary choices and the demand they create. However, the greater resistance among daily beef eaters highlights the deeply entrenched sociocultural and dietary habits that prioritize beef as a staple food.

To promote more sustainable food choices, comprehensive communication strategies must address both intrinsic and extrinsic barriers to change. Policymakers and health organizations should focus on developing strategies that not only inform the public about the environmental impacts of global beef production but also encourage them to take actionable steps. Understanding the motivations behind dietary changes, including environmental concerns, is important for designing effective approaches that can lead to long-term sustainability in food consumption.

Future research should experimentally evaluate the short- and long-term efficacy of environmental dietary change campaigns once implemented and identify the most effective strategies for sustaining changes in food choices and eating behaviors. Of note, a brief pause in beef consumption could also serve as a practical entry point for individuals to try dietary alternatives, providing exposure to new tastes and textures while reducing habitual dependence on beef. Compelling, evidence-based appeals that help translate awareness into action can support individuals in adopting more sustainable dietary habits. These shifts in consumption, when widely adopted, can contribute to climate change mitigation and planetary sustainability.

## Figures and Tables

**Table 1:** #NoBeefWeek acceptance rates.

Beef Eaters’ Responses		*n*	%
Likelihood of avoiding beef and its derivative products during #NoBeefWeek	Extremely likely	391	54.2
	Likely	156	21.6
	Unsure	58	8.0
	Unlikely	38	5.3
	Extremely unlikely	78	10.8
**All responses (beef eaters and beef avoiders)**		***n***	**%**
Likelihood of encouraging family and friends to avoid beef and its derivative products during #NoBeefWeek	Extremely likely	490	38.6
	Likely	356	28.0
	Unsure	199	15.7
	Unlikely	116	9.1
	Extremely unlikely	110	8.7

**Table 2:** Long-term beef consumption change intentions in connection with current beef intake frequencies.

Long-Term Intention to Change Own Beef Consumption Relative to Self-Reported Beef Intake Frequency	Stop My Consumption	Minimize My Consumption	Reduce My Consumption	Keep My Consumption Unchanged	WAGV ^1^	Total Responses *n*
	Percentage ^2^					
**Every day**	10.7	14.3	7.1	67.9	2.32	28
**Four to six times per week**	**3.3**	**14.8**	**32.8**	**49.2**	2.28	61
**Two to three times per week**	**7.5**	**25.4**	**42.2**	**24.9**	1.84	173
**Once a week**	**11.7**	**30.3**	**37.7**	**20.4**	1.67	162
**A couple of times a month or less**	**29.2**	**36.4**	**20.8**	**13.6**	1.19	264

^1^ Levels of change resistance: The ranking of beef intake frequencies from greatest to least resistance to long-term beef intake cutbacks is based on weighted averages calculated on the scale ‘stop my consumption’ (0), ‘minimize my consumption’ (1), ‘reduce my consumption’ (2), and ‘keep my consumption unchanged’ (3). ^2^ Bolded values represent significant differences (*p* < 0.05) in beef consumption change intentions between the various beef intake frequency groups.

## Data Availability

The data presented in this study are available on reasonable request from the corresponding author. The data are not publicly available due to ethical restrictions.

## Supplementary Materials

The following supporting information can be downloaded at https://www.mdpi.com/article/10.3390/foods14152620/s1: Table S1: General characteristics of the study participants; Table S2: Self-reported levels of difficulty for avoiding different beef products for at least one week; Table S3: Views about beef consumption in relation to human health and the environment; Table S4: Resistance levels to cut back beef intake relative to environmental views across all five related statements. Table S5: Levels of disagreement based on beef consumption frequency for each of the five environmental statements.

## Abbreviations

WAVG—weighted average. WHO—World Health Organization. COP—Conference of the Parties, overseen by the United Nations Framework Convention on Climate Change (UNFCCC). OECD—Organisation for Economic Co-operation and Development. FAO—Food and Agriculture Organization of the United Nations. COM-B—Capability (C), Opportunity (O) and Motivation (M) Model of Behavior Change (B).
