# Supplementary material for: Sustainability Views and Intentions to Reduce Beef Consumption: An International Web-Based Survey
Source: Foods. 2025 Jul 26;14(15):2620. doi: 10.3390/foods14152620 (PMC12346450; doi:10.3390/foods14152620)
Supplement: Supplementary file 1 [file foods-14-02620-s001.zip › Supplementary Table S1.pdf]

Table S1. General characteristics of the study participants.

Variables		*n*	%
**Age**	18-20	22	1.7
	21-25	81	6.1
	26-30	107	8.1
	31-35	125	9.4
	36-40	154	11.6
	41-45	159	12.0
	46-50	167	12.6
	51-55	167	12.6
	56-60	125	9.4
	61-65	90	6.8
	66-70	56	4.2
	70 or older	67	5.0
**Gender**	Male	344	26.1
	Female	964	73.1
	Other	1	0.1
	Prefer not to say	9	0.7
**Country of residence**	United Kingdom	315	27.0
	United States	315	27.0
	India	106	9.1
	Canada	62	5.3
	Australia	35	3.0
	France	34	2.9
	Spain	22	1.9
	Nigeria	19	1.6
	South Africa	18	1.5
	Pakistan	13	1.1
	Ireland	12	1.0
	New Zealand	12	1.0

	Variables	*n*	%
	Other 72 countries	204	17.6
**Current diet**	I only eat animal foods	13	1.0
	I only eat plant foods	253	19.0
	I predominantly eat plant foods, plus some animal foods	495	37.2
	I predominantly eat animal foods, plus some plant foods	96	7.2
	I eat a balance of plant foods and animal foods	475	35.7
**Consumption frequency of beef or its derivative products**	I don't eat beef or derivatives	306	28.7
	Every day	30	2.8
	4 to 6 times per week	69	6.5
	2 to 3 times per week	199	18.6
	Once a week	181	17.0
	A couple of times a month or less	283	26.5
**Increased interest in beef consumption and planetary health from completing the survey**	Yes, I'd like to know more	471	40.1
	A little	327	27.8
	No	378	32.1
**Being a nutrition or health professional**	Yes	508	43.1
	No	481	40.8
	Studying for it	191	16.2
