# Supplementary material for: Sustainability Views and Intentions to Reduce Beef Consumption: An International Web-Based Survey
Source: Foods. 2025 Jul 26;14(15):2620. doi: 10.3390/foods14152620 (PMC12346450; doi:10.3390/foods14152620)
Supplement: Supplementary file 1 [file foods-14-02620-s001.zip › Supplementary Table S2.pdf]

Table S2. Self-reported levels of difficulty for avoiding different beef products for at least one week.

Ranking of beef products from easiest to hardest to avoid consuming for at least one week		Extremely easy	Easy	Unsure	Difficult	Extremely difficult	WAGV*	Total responses *n*
		%						
1	Hamburgers	64.8	25.9	3.6	2.5	3.2	1.5	721
2	Meatballs	67.8	23.5	3.1	2.8	2.8	1.5	718
3	Pâté / spreads	78.4	15.2	2.7	2.1	1.7	1.5	718
4	Salami / pepperoni / salt-cured cuts	71.7	18.9	3.6	3.5	2.4	1.6	721
5	Sausages	70.5	19.5	4.0	3.2	2.8	1.6	718
6	Beef in wraps, sandwiches, sauce, stuffing, pies, etc.	67.9	21.1	3.7	4.4	2.9	1.7	722
7	Minced (ground) beef	53.6	28.0	8.2	6.4	3.9	1.7	719
8	Steaks / fillets / other cuts	58.1	25.5	5.3	6.2	5.0	1.8	723

* The ranking of beef products from easiest to hardest to give up for at least one week is based on weighted averages calculated on the scale ‘extremely easy’ (0), ‘easy’ (1), ‘unsure’ (2), ‘difficult’ (3), and ‘extremely difficult’ (4).
