# Supplementary material for: Sustainability Views and Intentions to Reduce Beef Consumption: An International Web-Based Survey
Source: Foods. 2025 Jul 26;14(15):2620. doi: 10.3390/foods14152620 (PMC12346450; doi:10.3390/foods14152620)
Supplement: Supplementary file 1 [file foods-14-02620-s001.zip › Supplementary Table S3.pdf]

Table S3. Views about beef consumption in relation to human health and the environment.

	Statements presented to participants	Strongly Agree	Slightly Agree	Neither Agree nor Disagree	Slightly Disagree	Strongly Disagree	Total responses
		%					*n*
1	Beef consumption negatively impacts planetary health	55.3	20.2	14.2	4.6	5.8	1,208
2	Beef consumption negatively impacts human health	45.5	24.1	15.3	7.7	7.4	1,210
3	Beef consumption is the leading cause of deforestation in the Amazon and other tropical forests	38.1	23.9	26.0	5.2	6.7	1,207
4	Beef consumption is one of the main causes of global climate change	35.0	23.1	22.5	8.1	11.4	1,205
5	Beef consumption results in more greenhouse gas emissions than plant-food consumption	53.0	18.4	17.2	4.9	6.5	1,205
6	The livestock industry is the biggest contributor of global greenhouse gas emissions from food production	46.1	23.8	18.7	5.1	6.4	1,207
