# Supplementary material for: Sustainability Views and Intentions to Reduce Beef Consumption: An International Web-Based Survey
Source: Foods. 2025 Jul 26;14(15):2620. doi: 10.3390/foods14152620 (PMC12346450; doi:10.3390/foods14152620)
Supplement: Supplementary file 1 [file foods-14-02620-s001.zip › Supplementary Table S4.pdf]

Table S4. Resistance levels to cut back beef intake relative to environmental views across all five related statements.

Long-term beef cutback intentions						
**Beef eaters' views across all five environmental statements**	Stop my consumption	Minimize my consumption	Reduce my consumption	Keep my consumption unchanged	Change Resistance Levels WAGV[1]	Total responses *n*
	%[2]					
Strongly agree	35.4	29.0	30.0	5.6	1.1	968
Slightly agree	14.6	33.1	40.7	11.7	1.5	883
Neither agree nor disagree	9.1	34.3	33.0	23.6	1.7	891
Slightly disagree	4.3	29.7	28.0	38.0	2.0	279
Strongly disagree	1.2	11.6	6.8	80.4	2.7	413
**Total**	16.6	30.6	29.4	23.4	-	3434

Long-term beef cutback intentions per environmental impact statement						
**Beef eaters' views on *'Beef consumption negatively impacts planetary health'***	Stop my consumption	Minimize my consumption	Reduce my consumption	Keep my consumption unchanged	Change Resistance Levels WAGV[1]	Total responses *n*
	%[3]					
Strongly agree	**34.3**	**28.0**	**32.2**	**5.5**	1.1	236
Slightly agree	**10.3**	**35.1**	**39.7**	**15.0**	1.6	194
Neither agree nor disagree	**9.4**	**34.5**	**31.7**	**24.5**	1.7	139
Slightly disagree	**0.0**	29.4	23.5	**47.1**	2.2	51
Strongly disagree	**0.0**	**7.5**	**1.5**	**91.0**	2.8	67

**Beef eaters' views on *'Beef consumption is the leading cause of deforestation in the Amazon and other tropical forests'***	Stop my consumption	Minimize my consumption	Reduce my consumption	Keep my consumption unchanged	Change Resistance Levels WAGV[1]	Total responses *n*
	%[3]					
Strongly agree	**39.4**	**31.0**	**25.8**	**3.9**	0.9	155
Slightly agree	**20.8**	**29.2**	**39.3**	**10.7**	1.4	178
Neither agree nor disagree	**6.2**	**35.1**	**35.1**	**23.6**	1.8	225
Slightly disagree	**1.8**	30.9	29.1	**38.2**	2.0	55
Strongly disagree	**1.4**	**8.1**	**6.8**	**83.8**	2.7	74

**Beef eaters' views on *'Beef consumption is one of the main causes of global climate change'***	Stop my consumption	Minimize my consumption	Reduce my consumption	Keep my consumption unchanged	Change Resistance Levels WAGV[1]	Total responses *n*
	%[3]					
Strongly agree	**39.5**	**27.1**	**29.5**	**3.9**	1.0	129
Slightly agree	**17.2**	**35.0**	**39.9**	**8.0**	1.4	163
Neither agree nor disagree	15.1	**34.4**	**33.9**	**16.7**	1.5	192
Slightly disagree	**4.0**	33.3	33.3	**29.3**	1.9	75
Strongly disagree	**2.4**	**15.1**	**11.9**	**70.6**	2.5	126

**Beef eaters' views on *'Beef consumption results in more green-house gas emissions***	Stop my consumption	Minimize my consumption	Reduce my consumption	Keep my consumption unchanged	Change Resistance Levels WAGV[1]	Total responses *n*
	%[3]					

than plant-food consumption'						
Strongly agree	**32.0**	**29.6**	**30.4**	**7.9**	1.1	253
Slightly agree	**10.9**	**34.6**	**41.7**	**12.8**	1.6	156
Neither agree nor disagree	**7.0**	**33.1**	**33.8**	**26.1**	1.8	157
Slightly disagree	**8.3**	29.2	22.9	**39.6**	1.9	48
Strongly disagree	**1.4**	**9.5**	**6.8**	**82.4**	2.7	74
**Beef eaters' views on *'The livestock industry is the biggest contributor of global greenhouse gas emissions from food production'***	Stop my consumption	Minimize my consumption	Reduce my consumption	Keep my consumption unchanged	Change Resistance Levels WAGV[1]	Total responses *n*
	%[3]					
Strongly agree	**35.4**	**29.2**	**30.3**	**5.1**	1.1	195
Slightly agree	**14.1**	**31.8**	**42.7**	**11.5**	1.5	192
Neither agree nor disagree	**7.9**	**34.3**	**29.8**	**28.1**	1.8	178
Slightly disagree	**8.0**	24.0	28.0	**40.0**	2.0	50
Strongly disagree	**0.0**	**15.3**	**2.8**	**81.9**	2.7	72

[1] Levels of change resistance: The ranking from least to greatest resistance to long-term beef intake cutbacks is based on weighted averages calculated on the scale 'stop my consumption' (0), 'minimize my consumption' (1), 'reduce my consumption' (2), and 'keep my consumption unchanged' (3).

[2] Aggregated data (weighted averages of answers) across all five environment-related statements.

[3] Bolded values represent significant differences ($p < 0.05$) in beef consumption change intentions among groups with differing environmental views.
