# Supplementary material for: Sustainability Views and Intentions to Reduce Beef Consumption: An International Web-Based Survey
Source: Foods. 2025 Jul 26;14(15):2620. doi: 10.3390/foods14152620 (PMC12346450; doi:10.3390/foods14152620)
Supplement: Supplementary file 1 [file foods-14-02620-s001.zip › Supplementary Table S5.pdf]

Table S5. Levels of disagreement based on beef consumption frequency for each of the five environmental statements.

Beef consumption negatively impacts planetary health

**Self-reported current beef intake**	Strongly Agree	Slightly Agree	Neither Agree nor Disagree	Slightly Disagree	Strongly Disagree	Total responses
	%[2]					*n*
Every day	14.3	14.3	7.1	3.6	60.7	28
4 to 6 times per week	**11.5**	23.0	**19.7**	**11.5**	**34.4**	61
2 to 3 times per week	**20.4**	**34.3**	**22.7**	**12.8**	**9.9**	172
Once a week	**37.7**	**32.1**	**24.1**	**3.1**	**3.1**	162
A couple of times a month or less	**48.7**	**24.5**	**18.1**	**6.0**	**2.6**	265
I don't eat beef or derivatives[1]	**74.9**	**14.0**	**8.6**	**1.4**	**1.1**	279

Beef consumption is the leading cause of deforestation in the Amazon and other tropical forests

**Self-reported current beef intake**	Strongly Agree	Slightly Agree	Neither Agree nor Disagree	Slightly Disagree	Strongly Disagree	Total responses
	%[2]					*n*
Every day	14.3	14.3	14.3	7.1	50.0	28
4 to 6 times per week	**1.6**	23.0	24.6	**13.1**	**37.7**	61
2 to 3 times per week	**12.7**	23.7	**38.2**	**14.5**	**11.0**	173
Once a week	**28.6**	27.3	**34.8**	**3.7**	**5.6**	161
A couple of times a month or less	**30.9**	28.3	**32.1**	**5.3**	**3.4**	265

I don't eat beef or derivatives[1]	**47.8**	24.8	**22.3**	**2.9**	**2.2**	278

Beef consumption is one of the main causes of global climate change

**Self-reported current beef intake**	Strongly Agree	Slightly Agree	Neither Agree nor Disagree	Slightly Disagree	Strongly Disagree	Total responses *n*
			%[2]			
Every day	3.6	14.3	14.3	7.1	60.7	28
4 to 6 times per week	**6.6**	**11.5**	29.5	9.8	**42.6**	61
2 to 3 times per week	**14.0**	20.4	27.3	**19.2**	**19.2**	172
Once a week	**21.6**	**26.5**	**29.0**	**8.0**	**14.8**	162
A couple of times a month or less	**24.7**	**28.1**	**29.3**	**8.0**	**9.9**	263
I don't eat beef or derivatives[1]	**45.7**	**25.2**	**19.8**	**6.5**	**2.9**	278

Beef consumption results in more greenhouse gas emissions than plant-food consumption

**Self-reported current beef intake**	Strongly Agree	Slightly Agree	Neither Agree nor Disagree	Slightly Disagree	Strongly Disagree	Total responses *n*
			%[2]			
Every day	14.3	3.6	10.7	10.7	60.7	28
4 to 6 times per week	**18.0**	**13.1**	**24.6**	**11.5**	**32.8**	61
2 to 3 times per week	**27.8**	**25.4**	**27.8**	**10.4**	**8.7**	173
Once a week	**37.7**	**27.2**	**24.7**	**4.3**	**6.2**	162
A couple of times a month or less	**48.7**	**22.3**	**19.6**	**4.9**	**4.5**	265

I don't eat beef or derivatives[1]	**68.8**	**15.6**	**10.9**	**3.3**	**1.5**	276

The livestock industry is the biggest contributor of global greenhouse gas emissions from food production

**Self-reported current beef intake**	Strongly Agree	Slightly Agree	Neither Agree nor Disagree	Slightly Disagree	Strongly Disagree	Total responses *n*
			%[2]			
Every day	14.3	10.7	14.3	3.6	57.1	28
4 to 6 times per week	**11.5**	**9.8**	**36.1**	**11.5**	**31.2**	61
2 to 3 times per week	**21.4**	**32.4**	**27.2**	**11.6**	**7.5**	173
Once a week	**32.1**	**31.5**	**24.7**	**4.3**	**7.4**	162
A couple of times a month or less	**36.0**	**28.8**	**25.0**	**5.7**	**4.6**	264
I don't eat beef or derivatives[1]	**61.2**	**22.7**	**11.2**	**3.6**	**1.4**	278

[1] Data from respondents who reported consuming animal foods while avoiding beef products.

[2] Aggregated data (weighted averages of answers) across all five environment-related statements.

[3] Bolded values represent significant differences ($p < 0.05$) in environmental views among groups with varying beef consumption frequencies, including no consumption.
